# Learnings from familial visual snow syndrome: A case series

**DOI:** 10.1111/head.70098

**Published:** 2026-04-14

**Authors:** Laura Weichsel, Nedelina Slavova, Franz Riederer, Adrian Scutelnic, Antonia Klein, Sophie De Beukelaer, André Schaller, Christiane Zweier, Christoph J. Schankin

**Affiliations:** ^1^ Department of Neurology, Inselspital, Bern University Hospital University of Bern Bern Switzerland; ^2^ Department of Human Genetics, Inselspital, Bern University Hospital University of Bern Bern Switzerland; ^3^ Department of Neurology, Bellevue Medical Group Centre for Migraine and Headache Zurich Switzerland

**Keywords:** aura, genetics, migraine, visual snow syndrome

## Abstract

Visual snow syndrome is a neurological condition defined by the core symptom of visual snow and various additional visual and nonvisual symptoms. It can be of significant impact for affected individuals and treatment is currently challenging. Following an index patient reporting a clustering of visual snow syndrome in his family, we identified a total of six affected family members in three generations (Graphical abstract). Intrafamilial variability was high with different phenotypes, ages of onset, and triggering events. This family provides insights into the spectrum of visual snow syndrome and suggests avoiding triggers in relatives of individuals with visual snow syndrome. Studying the genetic mechanisms in families with visual snow syndrome might be an important step toward new preventive and therapeutic insights.

**Abbreviations**
VS: visual snow
VSS: visual snow syndrome

## INTRODUCTION

Visual snow syndrome (VSS) presents with a consistent clinical phenotype across geographic populations.^1^ Its main symptom is the persistent perception of tiny flickering dots, resembling television static. Common additional visual symptoms include palinopsia, entoptic phenomena, photophobia, and nyctalopia.^2^, ^3^, ^4^, ^5^, ^6^, ^7^ Nonvisual systems may also be affected, with patients reporting tinnitus, concentration problems, learning difficulties, and anxiety—suggesting involvement of the auditory and limbic systems. The underlying pathophysiology remains unclear but is hypothesized to involve disrupted brain networks, including serotonergic, GABAergic, and glutamatergic systems.^8^, ^9^, ^10^ VSS is considered rare, yet it is relatively frequent compared to other diseases present from birth, with a reported prevalence of 1%–2.2% in a British and Italian study.^11^, ^12^ Still, a considerable number of individuals live with the condition without having ever been diagnosed.^12^ There are currently no evidence‐based effective pharmacological treatments.^13^, ^14^, ^15^, ^16^ This might be in part due to the lack of understanding the pathophysiological, including the genetic basis of VSS. To date, a familial form of VSS has not been described in the literature.

Here, we present a family with multiple individuals affected by VSS. This family offers the unique opportunity to investigate the clinical spectrum in a quite homogeneous genetic background.

## MATERIALS AND METHODS

This case presentation was conducted in accordance with the Declaration of Helsinki and reviewed by the ethics committee of the Canton of Berne (BASEC‐ID Req‐2025‐00534). Written informed consent for publication was obtained from the participating family members. During a routine consultation at our tertiary headache center, which specializes in VSS, a patient (index case, V:4) reported similar symptoms in several relatives. To assess the family history, we provided a checklist based on the International Classification of Headache Disorders 3rd edition VSS criteria and comorbidities, which the patient shared with family members. Some also agreed to semi‐structured telephone interviews. We collected information on symptom onset, symptom type, and comorbidities such as migraine, tinnitus, depression, and anxiety. Based on the data, relatives were categorized into three groups, with the presence of at least two visual symptoms, as required by the VSS diagnostic criteria, serving as the cutoff: (1) those meeting full International Classification of Headache Disorders 3rd edition VSS criteria, (2) those with at least two visual symptoms not meeting full criteria, and (3) those without relevant symptoms. Additional clinical data were extracted from the medical records.

## RESULTS

**FIGURE 1:**
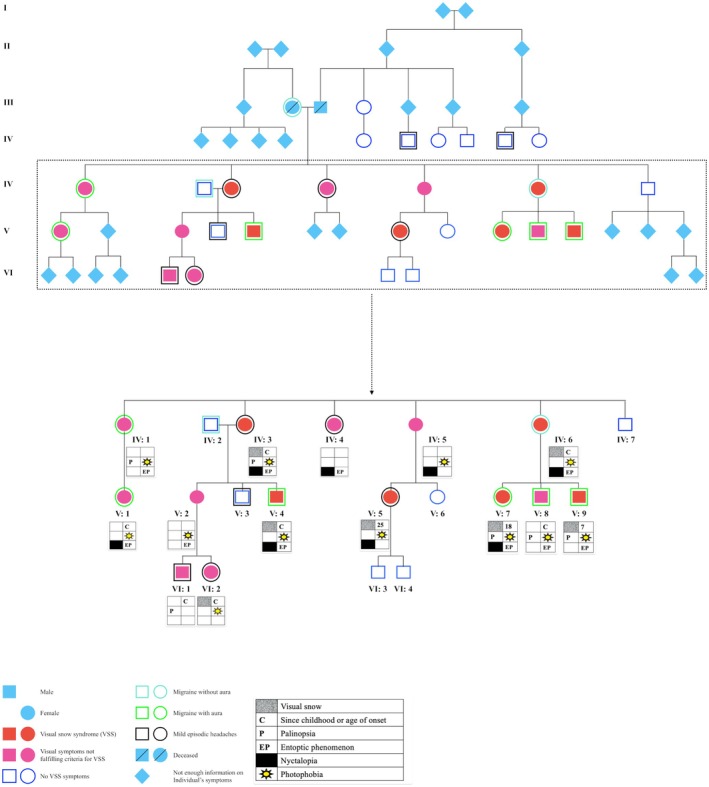
Pedigree of a family with several members experiencing visual snow syndrome (in red). Family members with at least two visual symptoms but not fulfilling the criteria of visual snow syndrome were marked pink. Individuals not affected are blue‐framed. Light green and mint green frames show individuals with migraine with or without aura and black frames stand for mild episodic headaches. Above, the whole family is displayed. Below, the three generations of family members we had a closer look at are shown. The specific symptom profiles of each subject were summarized in a comparative overview. [Color figure can be viewed at wileyonlinelibrary.com]

The pedigree (Figure 1) was developed together with the index patient (V:4) and includes responses from 31 individuals across four generations, aged from early childhood to nearly 100 years. VSS was identified in six individuals (marked in red), with visual snow (VS) reported in seven. Eight others reported visual symptoms not fulfilling VSS criteria (marked in pink). Nonparticipating members are represented as rhombi.

In the three most recent generations, detailed characterization revealed phenotypic variability with overlapping symptom patterns. Among all symptoms, VS was considered the most bothersome symptom in all six affected individuals and was often accompanied by nyctalopia and tinnitus. VS was mainly described as black‐and‐white (*n* = 5) or transparent (*n* = 1) dots, occasionally with color in addition (*n* = 2). Palinopsia occurred in both family members with VSS (*n* = 3) and without VSS (*n* = 3). Photophobia (*n* = 11) and nyctalopia (*n* = 8) were most frequent. Entoptic phenomena, especially floaters, were common in both, family members with VSS (*n* = 5) and without VSS (*n* = 4). Symptom patterns, including overlaps within parent–child pairs, are illustrated in the pedigree (Figure 1).

Migraine with aura (*n* = 6) and without aura (*n* = 2) were common comorbidities. Mild episodic headaches (*n* = 8), anxiety (*n* = 4), and depression (*n* = 1) were also reported. With the exception of two family members, tinnitus was not a characteristic finding in this family. One member experienced tinnitus after taking bupropion and the other member described episodic tinnitus associated with migraine attacks.

Symptoms were generally stable, with no increase in severity; in one case, they were perceived as less severe over time. However, symptoms appeared to wax and wane in relation to lighting conditions, tiredness, during episodes of illness or during migraine aura.

Certain characteristics are emphasized in the following six case descriptions of family members experiencing VSS (Table 1).

**TABLE 1:** Summary of the six cases of VSS including onset and possible triggers (pregnancy and stress) as well as additional visual symptoms and nonvisual symptoms.

Case	1	2	3	4	5	6
Sex	Male	Female	Female	Male	Female	Female
VS	VS	VS	VS	VS	VS	VS
Since C or age of onset	C	25	18	7	C	C
Trigger		Pregnancy	Stress, first migraine aura episode, sertraline			
Palinopsia			P	P	P	
Enhanced entoptic phenomena	EP		EP	EP	EP	EP
Nyctalopia	N	N	N		N	N
Photophobia	Ph	P	Ph	Ph	Ph	Ph
MwoA/MwA	MwA		MwA	MwA		MwoA
Tinnitus	T			T		
Anxiety		A	A	A	A	
Depression	D					

Abbreviations: A, anxiety; C, childhood; D, depression; EP, entoptic phenomenon; MwA, migraine with aura; MwoA, migraine without aura; N, nyctalopia; P, palinopsia; Ph, Photophobia; T, tinnitus; VS, visual snow; VSS, visual snow syndrome.

### Case 1 (index patient; V:4)

This man in his mid‐20s experiences lifelong VS. Initial symptoms occurred during ball games in overcast conditions and worsened notably during nighttime games. Initial concerns about learning difficulties in childhood were later attributed to impaired visuospatial perception. He reports tinnitus, migraine with and without aura since age 7, along with Alice in Wonderland syndrome.

### Case 2 (V:5)

This woman in her early 30s first experienced VS during pregnancy at age 25 years. VSS has remained constant without any fluctuations. Initial symptom‐related anxiety was treated with escitalopram, without impact on VSS.

### Case 3 (V:7)

This woman in her mid‐20s developed VSS symptoms around the age of 18 years, coinciding with a stressful period and initiation of sertraline treatment. Onset was accompanied by her first attack of migraine with aura. VS is most noticeable for approximately 1 hour outdoors on bright days. In addition to VSS, she has been diagnosed with anxiety and obsessive‐compulsive disorders.

### Case 4 (V:9)

This man in his early 30s recalled noticing VSS beginning at age 7 years. It became especially apparent when looking at a cloudy sky. VSS has become less intrusive over time. Beyond visual symptoms, he experiences anxiety, treated with sertraline. Migraine with visual aura started at the age of 26 years and currently occurs episodically. Tinnitus can occur occasionally during the end of a migraine.

### Case 5 (IV:3)

This woman in her early 60s reported lifelong VSS symptoms with a stable course. The most impairing symptoms are persistent VS and nyctalopia, particularly when driving at night.

### Case 6 (IV:6)

This woman in her late 50s is only slightly affected by visual symptoms and had just recently noticed having VSS. She perceives VS depending on light conditions.

## DISCUSSION

The main findings of this case series are: (1) VSS can occur in families, (2) in familial VSS, every generation can be affected by the main symptom VS, (3) VSS in affected family members differs in age of onset and phenotype, and (4) family members not fulfilling the criteria of VSS might experience additional visual symptoms that typically occur in VSS.

VSS has mainly been documented in sporadic cases, suggesting random occurrence through incidental triggers or *de novo* mechanisms, complicating the identification of an underlying etiology.^2^, ^4^ No family cases have been reported in the literature; nonetheless, 2.4%–10% indicated affected first‐degree relatives.^2^, ^17^ The observation of this large family with multiple affected members indicates a more homogeneous hereditary component, at least for this affected family. The transmission pattern may be compatible with autosomal dominant inheritance or mitochondrial DNA involvement. However, variability in symptom severity would also be compatible with a multifactorial cause.

Although previous studies found no endophenotypes in genetically unrelated, self‐diagnosed VSS cases,^4^ applying an endophenotype model in a future genetic study of a more genetically homogeneous cohort, such as the one described here, may help explain phenotypic variability in symptom patterns and severity.

Since the early reports on VSS, affected individuals have been grouped in those having the syndrome since early childhood and those who had later onset. It has been unclear if these were different clinical entities. Having both groups in a family with several affected family members suggests that there might be a common, likely genetic, basis, and the various ages of onset reflect rather a spectrum than a fundamental difference. Also, the hypothesis that early onset might be less bothersome because the individual had the visual symptoms for the entire life leading to habituation is not supported by this report (first case, V:4 in Figure 1).

The progression from isolated visual symptoms to the full syndrome has not been observed in our report, but detailed history could not be taken due to the retrospective and cross‐sectional design. With the limitation of a recall bias, triggers leading to the outbreak of the full syndrome have been identified in two family members. In one case, the onset of VSS occurred abruptly during pregnancy, another during a stressful period. Associations were also noted between symptom onset and serotonergic medications or bupropion, consistent with previous case reports.^18^ In the remaining cases, the exact time of onset could not be recalled.

These findings are important for the counseling of patients in daily routine: it is important to discourage affected family members from using recreational drugs, given the risk of symptom aggravation.^19^ Also, their so‐far nonaffected relatives might be at risk to experience new‐onset of VSS on certain triggers.

Similarly, the episodic occurrence of VS (e.g., for 1 hour on a fair‐weather day or in the dark [three family members]), broadens the spectrum of VS and may indicate susceptibility to future symptom persistence. An episodic manifestation of VSS during migraine aura episodes (one individual) has also been reported.^20^, ^21^

Comorbid migraine (both with and without aura) and anxiety disorders were common in this family, aligning with findings in sporadic VSS.^22^, ^23^, ^24^ Because migraine is considered a risk factor for VSS, a shared pathophysiological mechanism has been proposed. However, the presence of VSS in people without migraine within the investigated family suggests that the two conditions may overlap, but not necessarily share a common basis.

The limitations of this case series are mainly its retrospective design with data coming from clinical routine. Still, it is the description of a large family with VSS, possibly pointing to a genetic cause. In the light of currently having no pharmacological treatment, studying the genetic background of VSS might be promising. Furthermore, this is only one family with a limited number of affected family members.

## CONCLUSION

Our findings suggest a genetic contribution to VSS within this family and highlight a broad clinical spectrum with variable onset and symptom combinations. Identifying and avoiding triggers may help reduce risk, and these insights could guide future genetic research and therapeutic strategies.

## AUTHOR CONTRIBUTIONS

**Laura Weichsel:** Conceptualization; writing – original draft; writing – review and editing; visualization; data curation; investigation; formal analysis. **Nedelina Slavova:** Writing – review and editing; formal analysis. **Franz Riederer:** Writing – review and editing; formal analysis. **Adrian Scutelnic:** Writing – review and editing; formal analysis. **Antonia Klein:** Writing – review and editing; formal analysis. **Sophie De Beukelaer:** Writing – review and editing; formal analysis. **André Schaller:** Writing – review and editing; formal analysis. **Christiane Zweier:** Writing – review and editing; visualization; formal analysis; conceptualization. **Christoph J. Schankin:** Supervision; data curation; writing – review and editing; writing – original draft; visualization; conceptualization; investigation; formal analysis.

## FUNDING INFORMATION

No funding was received toward this work, but some of the authors received funding by Visual Snow Initiative, Eye on Vision Foundation, Bangerter‐Rhyner Foundation, Visual Snow Syndrome Germany e.V., and Baasch Medicus Foundation.

## CONFLICT OF INTEREST STATEMENT

**Laura Weichsel, Nedelina Slavova, Franz Riederer, Adrian Scutelnic, Antonia Klein, Sophie De Beukelaer, André Schaller,** and **Christiane Zweier** declare no conflicts of interest. **Christoph J. Schankin** reports fees for consulting, advisory boards, speaker activities, and travel support for/from Abbvie, Almirall, Amgen, Eli Lilly, Lundbeck, Novartis, Pfizer, TEVA Pharmaceuticals, MindMed, Grünenthal; he is part‐time employee at Zynnon and Cefalognos; he received research grants from the German Migraine and Headache Society, Eye on Vision Foundation, Lundbeck, Swiss Heart Foundation, Teva Pharmaceuticals, Visual Snow Syndrome Germany e.V., Visual Snow Initiative, and Baasch Medicus Foundation.

## Data Availability

The data that support the findings of this study are available from the corresponding author on reasonable request and with ethics approval. Original data are not publicly available due to restrictions to protect the privacy of the family.
